# Optimization of Activated Rubber Asphalt Production Parameters Based on Rheological Properties and Multi-Index Evaluation

**DOI:** 10.3390/ma18153712

**Published:** 2025-08-07

**Authors:** Jing Zhao, Xiangqing Zhao, Bo Li, Yongning Wang, Huan Zhao, Kai Kang

**Affiliations:** 1 China Railway Investment Group Co., Ltd., Beijing 100195, China; 18813098377@163.com; 2 Northwest Research Institute Co., Ltd. of C.R.E.C., Lanzhou 730070, China; zhaoxiangqing2005@163.com; 3 National and Provincial Joint Engineering Laboratory of Road & Bridge Disaster Prevention and Control, Lanzhou Jiaotong University, Lanzhou 730070, China; 13679424751@163.com (Y.W.); axi0011@163.com (H.Z.); 4 Gansu Province Traffic Investment Management Co., Ltd., Lanzhou 730030, China

**Keywords:** road engineering, activated rubber asphalt, process parameters, rheological properties, RSR model theory, gray correlation theory

## Abstract

This study presents a method to more reasonably control the quality performance of activated rubber asphalt by microwave activation. Different activated rubber asphalt preparation process parameters (reaction temperature, stirring rate, and reaction time) were selected to explore the influence of different process parameters on the macroscopic properties of rubber asphalt, and a multi-indicator evaluation model was set up using the theoretical method of the RSR model to determine the optimal production process parameters. The results showed that reaction temperature had the strongest influence (gray correlation > 0.85) among production parameters, followed by stirring rate and reaction time. The optimal parameters identified were a reaction temperature of 220 °C, a stirring rate of 1000 rpm, and a reaction time of 120 min, under which the viscosity–temperature sensitivity decreased by approximately 18%, and the rutting factor (G*/sinδ) increased by over 20%, indicating significant improvements in rheological stability and high-temperature performance. The integrated evaluation approach provided reliable and practical guidance for producing high-performance activated rubber asphalt.

## 1. Introduction

With the rapid development of the automobile industry, China’s automobile production and ownership are also increasing year by year, which produces a large number of waste tires, causing great pressure on the environment. In order to solve the increasingly serious black pollution problem, the use of waste rubber powder to modify asphalt, which can produce a better modification effect, can effectively reduce environmental pollution. However, in actual production, storage, transport, use, and other processes, rubber asphalt easily segregates, and its high viscosity is the main reason that limits its wide application. In order to improve the compatibility between rubber powder and matrix asphalt and the performance of rubber powder, researchers have activated and then modified rubber powder, which can effectively improve the above problems by increasing the surface activity of rubber powder [1].

The current activation methods of rubber powder include physical, chemical, and biological methods [2]. Xie et al. [3,4] used acrylamide to activate waste rubber powder, and their results showed that the activation of rubber effectively improved its low-temperature performance and storage stability. Li et al. [5] used hydrogen peroxide for the activation of waste rubber powder, and their results showed that the high-temperature stability of rubber asphalt after activation and the elastic recovery capacity of the activated rubber asphalt significantly declined. Liu et al. [6] proposed a combination of microwave activation and chemical activation methods, and their results showed that the storage stability of waste rubber powder-modified asphalt significantly improved, and the activated modified asphalt had better resistance to cracking, rutting, and fatigue. Shahrzad et al. [7] prepared activated rubber powder by a hybrid method of microwave treatment and biomodification, and the stability and low-temperature properties of the modified asphalt were significantly improved. Szerb et al. [8] used organosilanes and fatty acids to activate the gum powder and found that the viscoelasticity and compatibility of the modified asphalt were significantly improved.

The effect of activated rubber obtained by different activation methods on the high-temperature performance of asphalt varies, but it is generally accepted that the activation of rubber reduces the high-temperature performance of asphalt. Li et al. [9] compared the performance difference between activated and untreated rubber powder-modified asphalt, and they showed that the activated rubber powder-modified asphalt has better low-temperature performance; however, the activation of rubber powder weakened its particle core; the dissolution process was accompanied by a chemical reaction; the high-temperature performance of rubber powder was insufficient, and adding an appropriate compatibilizer enhanced the interaction between the rubber powder and asphalt. Dong et al. [10] used three-layer twin-screw shear for the activation of rubber powder, and their results showed that the activated rubber-modified asphalt derived from this method exhibited good compatibility and did not have a negative impact on other properties; however, the activation process of high-temperature extrusion reduced the high-temperature performance of rubber asphalt. Some studies have also shown that the activation of rubber powder has a positive effect on the high-temperature performance of asphalt. Ibrahim et al. [11] used the γ-ray activation of rubber powder, and they showed that the activation of rubber powder obtained by asphalt modification can improve the compatibility between the two; the high- and low-temperature performances of asphalt were improved, and the modified asphalt exhibited a better high-temperature rutting resistance.

Activated rubber asphalt has been recognized for its excellent rheological properties, improved durability, and environmental benefits in road engineering applications. However, its actual engineering applications still face serious challenges, especially the uncertainties in key production parameters such as reaction temperature, mixing rate, and reaction duration, which seriously affect the performance of asphalt. Previous studies have mainly focused on rubber content or simple blending methods, but they lack the systematic optimization and comprehensive evaluation of combined production parameters, resulting in inconsistent asphalt quality.

In view of these problems, there is an urgent need to clarify the influence mechanism of key production parameters on the rheological properties of ARA and to determine the optimal production conditions. In addition, conventional evaluation methods often fail to integrate multiple rheological indicators, which may lead to biased or incomplete evaluation results. Therefore, in this study, 28 different activated rubber asphalt samples were used as experimental materials, and the rheological properties of activated rubber asphalt were measured by changing reaction temperature, stirring speed, and reaction time. The optimal combination of process parameters for activated rubber powder-modified asphalt was selected by using an RSR model. The results can provide the relevant technical support for the production process selection and engineering application of twin-screw-activated rubber powder-modified asphalt.

## 2. Experimental Design

### 2.1. Materials and Methods

#### 2.1.1. Raw Materials

The matrix asphalt was SK90# matrix asphalt, made in Gansu Luqiao Shanjian Technology Co., Ltd, Lanzhou, China. Each sample was tested three times, and the average was taken as the final result, and the key technical indexes of virgin asphalt are shown in Table 1.

**Figure 1:**
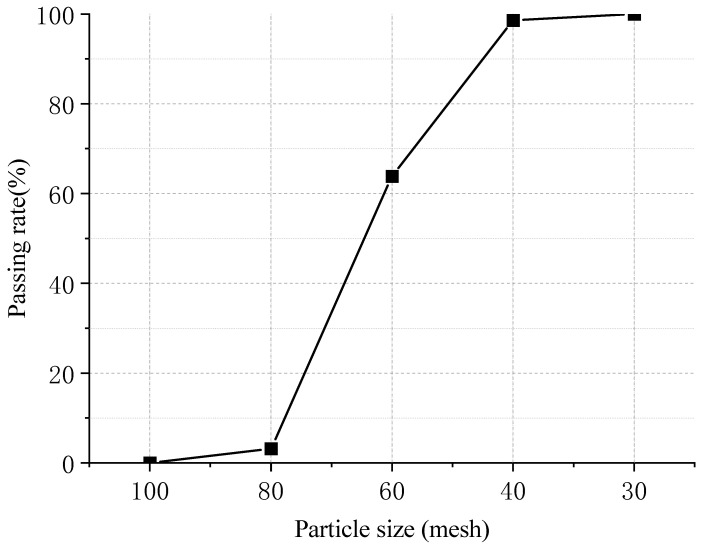
Gradation curve of rubber powder.

The rubber powder was waste tire rubber powder produced by China Gansu Luqiao Shanjian Technology Co., Ltd., and the rubber powder production process was carried out at the ambient temperature, with a maximum particle size of 40 mesh. Its gradation curve is shown in Figure 1, and the test results of the physical and chemical indexes of the rubber powder are shown in Table 2 and Table 3, respectively.

#### 2.1.2. Sample Preparation

(1) Preparation of activated rubber powder

First, the waste tire rubber powder was placed in a 60 °C oven for drying until the rubber powder was completely dried and the mass was constant. Then, the rubber powder was activated by a microwave device. The power of the microwave device was 800 W, and the activation time was 90 s, that is, the rubber powder was microwave-activated with a work of 72,000 J. During activation, specialized equipment was used to evenly spread the adhesive powder to ensure that its energy was evenly transmitted. The mass of activated rubber powder was 500 g.

(2) Preparation of rubber asphalt

A total of 500 g of matrix asphalt was heated to a completely flowing state and was poured into an iron reaction tank, and the asphalt was quickly heated to the specified temperature. Then, the iron reaction tank was placed in a constant-temperature magnetic heating stirrer. The temperature of this equipment could be automatically adjusted between 20 and 280 °C with an accuracy of 0.1 °C. The stirring rate was 0–10,000 rpm. Then, the rubber powder was first stirred slowly and then adjusted to the set rotation speed for stirring. After the stirring was completed, the rubber asphalt was taken out and sealed for storage for testing.

#### 2.1.3. Main Experimental Methods

(1) Brookfield viscosity test

Currently, the most commonly used viscosity test for rubber asphalt is the Brookfield viscosity test. In this study, the optimal process parameters for the viscosity test of activated rubber asphalt are as follows: a temperature of 180 °C, a 27# rotor, an asphalt mass of 12.5 g, and a rotation speed of 20 revolutions per minute. All tests must ensure that the torque is between 10 and 98% [11]. Due to the unstable characteristics of the viscosity test of rubber asphalt, the rotor was first placed in the asphalt sample and rotated for 15 min; then, the reading was taken after the viscosity result remains stable. Six test values were read every 10 s; then, the average value was taken as the viscosity test value. The equipment was provided by Brookfield DV2T, the United States.

(2) DSR test

The main indexes used for evaluating asphalt performance by the DSR test were complex modulus G*, phase angle δ, and rutting factor G*/sinδ. The equipment was provided by TA Instruments, the United States.

In research from the SHRP program in the United States, the rheological properties of activated rubber asphalt were characterized by DSR, and the temperature sensitivity of activated rubber asphalt was characterized by the complex modulus index method (GTS). GTS uses DSR to characterize the temperature sensitivity of activated rubber asphalt in the medium- and high-temperature ranges. The calculation formula of the complex modulus index method is as follows:

(1) $$\mathrm{lg}\mathrm{lg}G^{*}=GTS\cdot \mathrm{lg}T+C$$

where G* denotes the complex modulus, Pa; *GTS* denotes the complex modulus index; *T* denotes the test temperature, K; *C* denotes a constant.

When the absolute value of *GTS* is larger, it indicates that the temperature sensitivity of asphalt in this temperature range is lower; otherwise, it is higher.

### 2.2. Experimental Scheme

To determine the production process parameters of activated rubber asphalt, different process parameters were set. Referring to the research results of the US TRB [12], different preparation temperatures (160 °C, 190 °C, and 220 °C), stirring times (15 min, 60 min, 120 min, and 240 min), and stirring rates (1000 rpm, 2000 rpm, and 3000 rpm) were selected. The rubber powder content in all samples was 10%, and the matrix asphalt was SK90# matrix asphalt. A total of 7 groups of 28 different activated rubber asphalt samples were designed. The specific process parameter design scheme is shown in Table 4.

## 3. Results and Discussion

### 3.1. Influence of Process Parameters on Viscosity of Activated Rubber Asphalt

**Figure 2:**
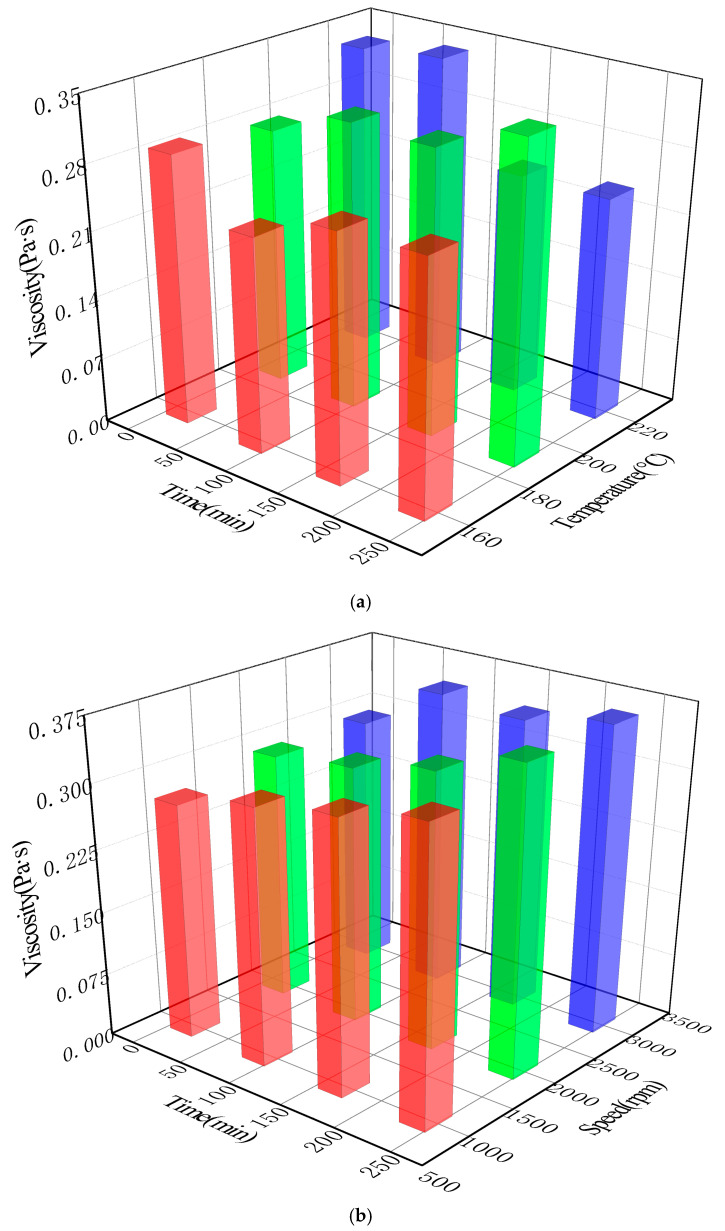
Influence of different process parameters on viscosity of activated rubber asphalt: (**a**) preparation temperature and (**b**) stirring rate.

Figure 2 shows the test results of the influence of different process parameters on the viscosity of activated rubber asphalt.

It can be seen from Figure 2 that different process parameters have different degrees of influence on the viscosity of activated rubber asphalt. The influence laws of different process parameters on the viscosity of activated rubber asphalt were analyzed, and are described in the following section.

#### 3.1.1. Preparation Temperature

Taking 1#, 3#, and 6# in Table 4 as examples, at lower temperatures of 160 °C and 190 °C and with a stirring rate of 1000 rpm, the viscosity of rubber asphalt gradually increases with time. This is shown in Figure 2. Rubber powder absorbed the light components in asphalt, resulting in its swelling, which increased the relative proportion of heavy components in asphalt, thus significantly improving the viscosity of the modified asphalt [12]. This indicates that, at lower temperatures, extending the reaction time is conducive to the swelling reaction of rubber asphalt. However, at a higher temperature (such as 220 °C), the viscosity of the activated rubber asphalt first increases and then decreases with the increase in reaction time. The extension of stirring time causes the viscosity of rubber asphalt to first increase and then decrease, reaching a peak at 60 s. This may be because the rubber powder undergoes desulfurization and degradation reactions with the increase in reaction time, resulting in the weakening of the cross-linking effect of rubber powder and thus reducing the viscosity of rubber asphalt.

#### 3.1.2. Stirring Rate

The influence of stirring rate on the viscosity of activated rubber asphalt was small (taking 1#, 3#, and 6# in Table 4 as examples, at a stirring temperature of 220 °C). When the stirring rate was 1000 rpm and 2000 rpm, it had no significant influence on the asphalt viscosity. However, when the reaction time was 240 min, the viscosity of rubber asphalt was significantly higher than that in other cases. When the stirring rate was 3000 rpm, the viscosity of rubber asphalt changed little in the initial stage of the reaction, but after 60 min, its viscosity significantly increased. With the increase in stirring rate, the viscosity remained relatively stable. This may be because the faster the stirring rate, the greater the external input energy, resulting in the rubber powder absorbing the oil in the asphalt to reach a saturated state. Subsequently, with the increase in reaction time, the viscosity also remained relatively stable.

### 3.2. Influence of Process Parameters on Viscoelasticity of Activated Rubber Asphalt

#### 3.2.1. Complex Modulus G* and Phase Angle δ

**Figure 3:**
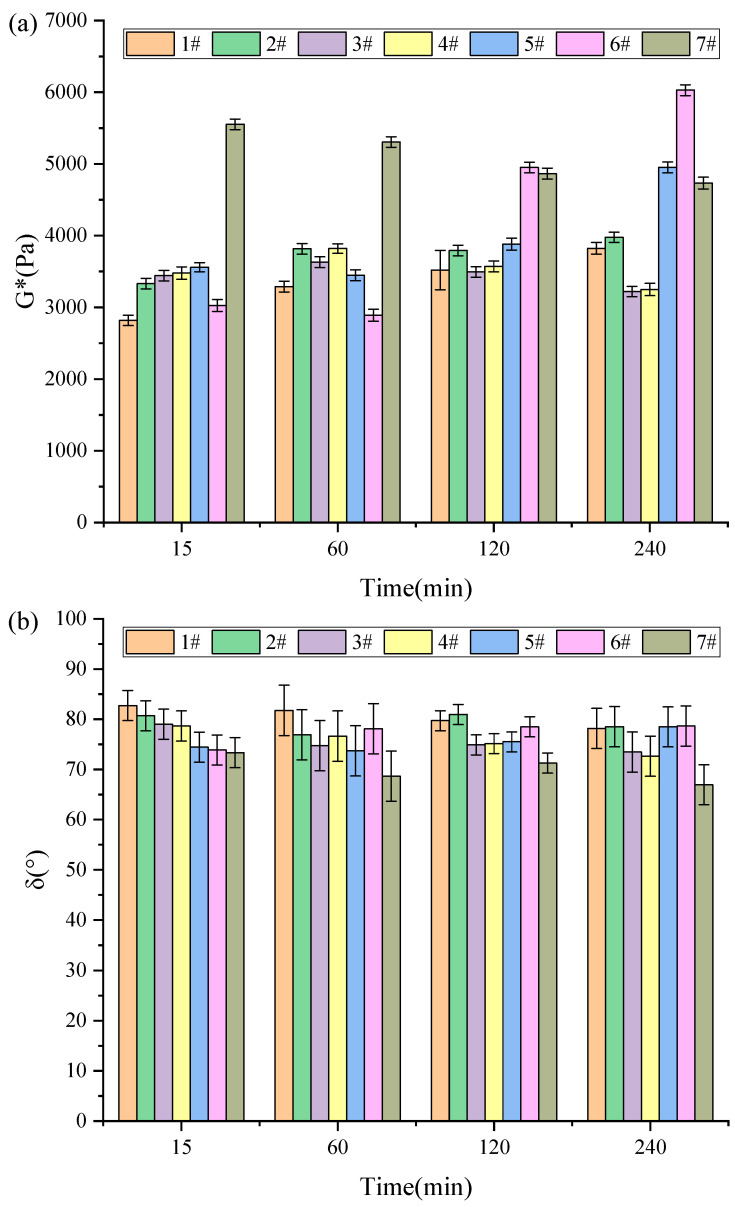
Results of G* (**a**) and δ (**b**) tests of activated rubber asphalt.

Figure 3 shows the complex shear modulus and phase angle of activated rubber asphalt under different process parameter conditions. It can be observed that, compared to the corresponding base asphalt, the overall rheological performance of activated rubber asphalt is significantly improved.

As shown in Figure 3a, the complex shear modulus of activated rubber asphalt steadily increases with the continuous increase in reaction time, while the phase angle gradually decreases. At 160 °C, the stirring speed has a limited effect on the rheological properties of activated rubber asphalt. Figure 3b further reveals how stirring speed and reaction time jointly affect the rheological parameters of activated rubber asphalt at 190 °C. All samples reached the highest complex modulus after 60 min of reaction. With the extension of reaction time, the complex modulus shows a decreasing trend, while the phase angle slightly decreases with the increase in stirring time. This phenomenon is mainly attributed to the swelling effect of the light components in the asphalt on the rubber powder, which becomes more significant with the increase in reaction time and stirring speed at lower temperatures (160 °C and 190 °C), thereby enhancing the rheological properties of activated rubber asphalt.

Figure 3b shows that, at the high temperature of 220 °C, the complex shear modulus of asphalt remains unchanged in the early stages of the reaction and then significantly increases in the later stages, while the phase angle gradually increases and eventually stabilizes. This change may be due to the rubber powder particles absorbing the light components from the asphalt and reaching saturation in the high-temperature environment. Subsequently, the rubber powder undergoes desulfurization and degradation during the reaction process, and these chemical changes lead to a significant improvement in the performance of rubber asphalt [12].

In summary, to prepare high-performance activated rubber asphalt, increasing the stirring speed or extending reaction time at lower temperatures (160 °C and 190 °C) can stabilize it. At higher temperatures (220 °C), activated rubber asphalt reaches a saturated state with a shorter reaction time or a lower stirring speed.

#### 3.2.2. G*/sinδ and Failure Temperature

**Figure 4:**
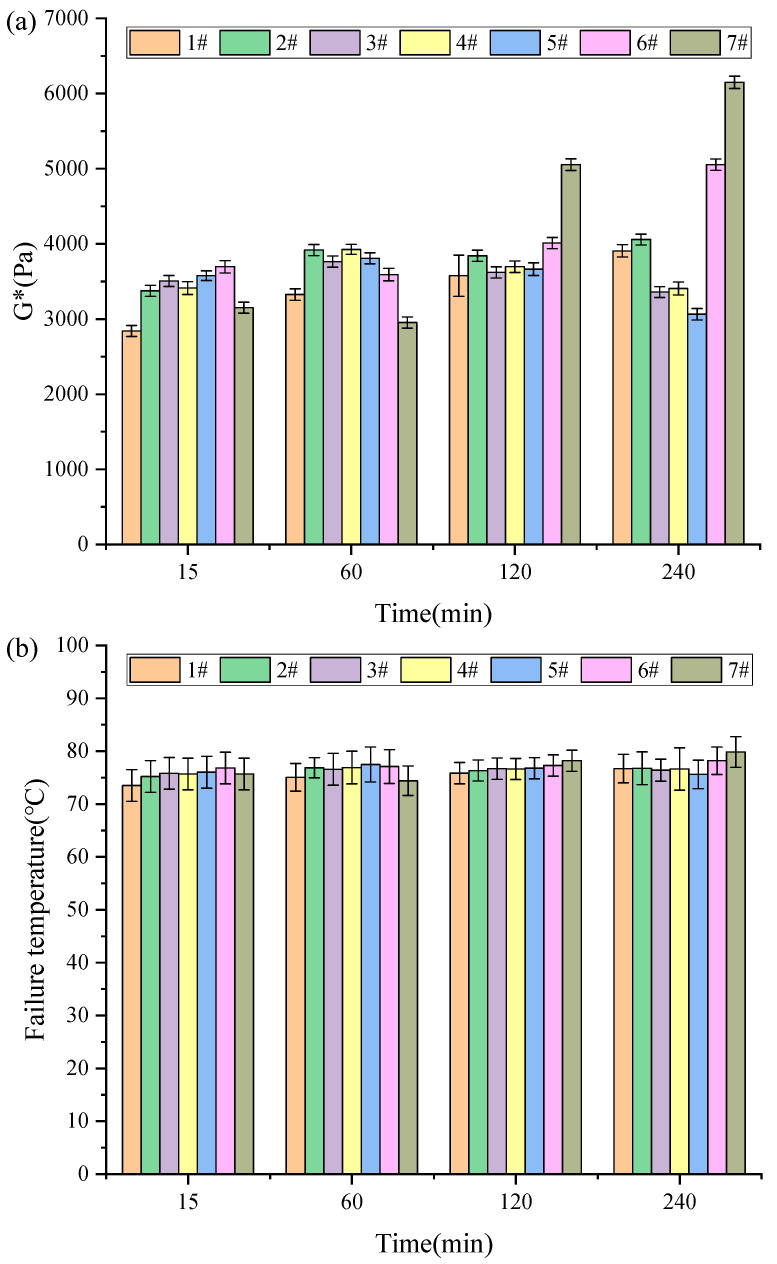
Different activated rubber asphalt rutting factor (**a**) and failure temperature (**b**) test results.

Figure 4 presents the rutting factor (G*/sinδ) and failure temperature test results for different activated rubber asphalts. G*/sinδ and failure temperature are important parameters for evaluating the rutting resistance and high-temperature grade of asphalt pavements. According to the Strategic Highway Research Program (SHRP), the high-temperature rutting factor of base asphalt should be greater than 1.0 kPa [13].

As shown in Figure 4a, the rutting factor of activated rubber asphalt follows the same trend as its complex modulus under different process parameters. At lower temperatures, the effects of stirring speed and reaction time on the rutting factor are minimal. However, at higher temperatures, stirring speed and reaction time have a significant impact on the rutting factor. As shown in Figure 4b, the failure temperature of activated rubber asphalt reaches 72 °C, indicating that the high-temperature performance of activated rubber asphalt is excellent.

#### 3.2.3. Effect of Process Parameters on High-Temperature Sensitivity of Activated Rubber Asphalt

**Figure 5:**
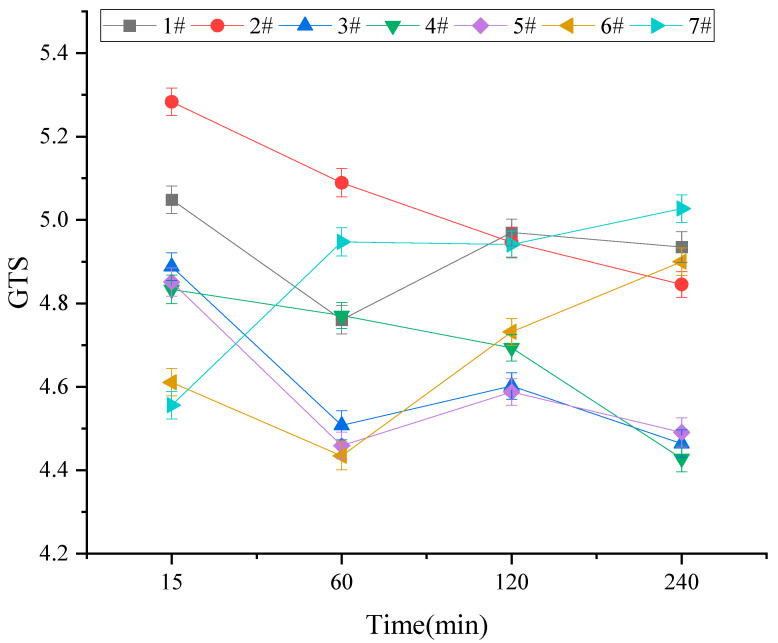
GTS test results of different activated rubber asphalt.

The high-temperature rheological properties of activated rubber asphalt were analyzed using Dynamic Shear Rheometer (DSR) testing at temperatures of 58 °C, 64 °C, 70 °C, 76 °C, and 82 °C. The high-temperature sensitivity of activated rubber asphalt was then calculated. The Global Temperature Sensitivity (GTS) results for different activated rubber asphalt samples are presented in Figure 5. The GTS value represents the sensitivity of asphalt to temperature changes under high-temperature conditions. A higher GTS value indicates poorer high-temperature sensitivity, while a lower value suggests better performance.

From Figure 5, it can be observed that the GTS values of activated rubber asphalt are significantly lower compared to those of the base asphalt, indicating a notable improvement in the high-temperature stability of the activated rubber asphalt. At lower reaction temperatures (160 °C and 190 °C), the GTS values generally increase with longer reaction times or higher stirring rates. However, the GTS values at 190 °C are slightly lower than those at 160 °C. These results demonstrate that, at lower reaction temperatures, the high-temperature rheological performance of activated rubber asphalt improves with the increase in reaction time, reaction temperature, and stirring rate. However, when the temperature reaches 220 °C, the GTS values of the activated rubber asphalt increase with the increase in stirring time, indicating weaker stability under high-temperature conditions.

#### 3.2.4. Gray Correlation Analysis

The gray relational analysis method is employed to investigate the impacts of reaction temperature, stirring time, and stirring rate of activated rubber asphalt on the rheological properties of rubber asphalt. Considering the rutting factor, failure temperature, viscosity, and GTS of activated rubber asphalt as evaluation indicators, this study examines the effects of stirring time, stirring rate, and reaction temperature on each evaluation indicator.

The gray relational degree method analyzes the primary and secondary relationships of the impacts of various test factors on the test results in the experiment through calculating the gray relational degree. Specifically, the larger the gray relational degree value, the more significant the influence of the selected factor on the test results. Conversely, the smaller the gray relational degree value, the smaller its influence on the test results. In the actual application process, the main influencing factors of the test results or the product production process can be controlled to improve product production efficiency and ensure product production quality. Its main calculation process is as follows:

There are m sub-sequences:

(2) $$\begin{array}{ccccc} t & x_{1}^{\left(t\right)} & x_{2}^{\left(t\right)} & \ldots & x_{n}^{\left(t\right)}\\ 1 & x_{1}^{\left(1\right)} & x_{1}^{\left(1\right)} & \ldots & x_{1}^{\left(1\right)}\\ 2 & x_{1}^{\left(2\right)} & x_{1}^{\left(2\right)} & \ldots & x_{1}^{\left(2\right)}\\ \vdots & \vdots & \vdots & & \vdots \\ m & x_{1}^{\left(m\right)} & x_{1}^{\left(m\right)} & \ldots & x_{1}^{\left(m\right)} \end{array}$$

For example, $\left\{x_{1}^{\left(0\right)}\left(t\right)\right\},\left\{x_{2}^{\left(0\right)}\left(t\right)\right\},\ldots ,\left\{x_{m}^{\left(0\right)}\left(t\right)\right\},t=1,2,\ldots ,N$. In addition, the time series (the mother sequence) is assumed to be $\left\{x_{0}^{\left(0\right)}\left(t\right)\right\},t=1,2,\ldots ,N$.

Considering the time series as the mother sequence and the m influencing factors as the sub-sequences, data transformation is performed. In this paper, the method of mean-centering is adopted based on the original data matrix. Then, the correlation coefficient is calculated according to Equation (3), and the degree of correlation is obtained according to Equation (4). Finally, sorting is performed according to the magnitude of the calculated degree of correlation, to obtain the primary and secondary relationships of the factors affecting the mother sequence.

(3) $$L_{0i}\left(k\right)=\frac{\Delta_{\min}+\rho \Delta_{\max}}{\Delta_{0i}\left(k\right)+\rho \Delta_{\max}}$$

In the above formula, $\Delta_{0i}\left(k\right)$——k denotes the absolute difference between the two compared sequences at a particular time, that is, $\Delta_{0i}\left(k\right)$=$\left|\Delta_{0}\left(k\right)-\Delta_{i}\left(k\right)\right|\left(1\leq i\leq m\right)$; $\Delta_{\max}$ and $\Delta_{\min}$ denote the maximum and minimum values of the absolute differences in each compared sequence at a particular time, respectively; ρ denotes the distinguishing coefficient, and 0<ρ<1. In general, it can take values from 0.1 to 0.5. In this paper, *ρ* = 0.5.

(4) $$\gamma_{0i}=\frac{1}{N}\sum_{k=1}^{N}L_{0i}\left(k\right)$$

In the above formula, $\gamma_{0i}$ represents the degree of correlation between the sub-sequence and the mother sequence, and N represents the length of the sequence.

Based on the aforementioned calculation method, reaction temperature, stirring time, and stirring speed are adopted as the sub-sequences for gray correlation analysis. Meanwhile, viscosity, the rutting factor, failure temperature, and GTS are taken as the mother sequences. Subsequently, the influence of each sub-sequence on the mother sequences is explored.

Table 5 shows the statistical results of the test values of various evaluation indicators of activated rubber asphalt under different process parameter conditions. Each group of activated rubber asphalt is numbered as 1, 2, 3, and 4, respectively. For example, the four samples in Group 1# are numbered as 1#-1, 1#-2, 1#-3, and 1#-4.

The gray correlation degree values between each evaluation index and the reaction parameters are calculated according to the calculation methods shown in Formulas (3) and (4), respectively, as presented in Table 6.

Table 6 shows that the main influencing factors of the evaluation indexes, sorted by their order of importance, are temperature, stirring rate, and reaction time. Additionally, the table clearly indicates that temperature is the primary factor influencing each index. Its gray correlation degree values for viscosity, the rutting factor, GTS, and failure temperature are 0.8638, 0.8686, 0.8539, and 0.8851, respectively. Therefore, temperature is the most crucial factor affecting the performance of activated rubber asphalt. The influencing degrees of stirring rate and reaction time are relatively small and similar to one another. Therefore, in the production and preparation processes of rubber asphalt, we need to control the temperature as accurately as possible to ensure the rationality of the reaction temperature. In addition, the mixing time and reaction rate should be selected based on the actual situation on-site and economic factors.

### 3.3. Optimization of Rubber Asphalt Production Parameters Based on RSR Model

#### 3.3.1. Basic Principles of Parameter Optimization Based on RSR Model Theory

The RSR model can simultaneously capture the sequential dynamics of user behavior and the relationship dependencies between items, thereby achieving more accurate dynamic preference prediction and recommendation results. The core concept of the RSR method lies in ranking the evaluation indicators and using their average value as the evaluation criterion. This method is applicable to the comprehensive evaluation and ranking of indicators with different measurement units, thus optimizing parameter selection in the rubber asphalt production process. The calculation steps of the RSR method are as follows:

(1) Construct the matrix

Set the number of evaluation objects to *n* and the number of evaluation indicators to m, and establish an (*n × m*) matrix to organize the data.

(2) Compile the rank matrix

In this study, the non-integer rank-sum ratio method is used to construct the rank matrix. By establishing a quantitative linear correspondence to link ranks with original indicator values, it effectively solves the problem of potential quantitative information loss of original indicator values during the RSR-based ranking process.

For positive indicators:

(5) $$R_{ij}=1+(n-1)\cdot \frac{X_{ij}-\min(X_{1j},X_{2j},\ldots ,X_{nj})}{\max(X_{1j},X_{2j},\ldots ,X_{nj})-\min(X_{1j},X_{2j},\ldots ,X_{nj})}$$

For negative indicators:

(6) $$R_{ij}=1+(n-1)\cdot \frac{\max(X_{1j},X_{2j},\ldots ,X_{nj})-X_{ij}}{\max(X_{1j},X_{2j},\ldots ,X_{nj})-\min(X_{1j},X_{2j},\ldots ,X_{nj})}$$

(3) Calculate the rank-sum ratio:

(7) $$RSR_{i}=\frac{1}{n\times m}\sum_{j=1}^{m}R_{ij}$$

where R_ij_ denotes the rank of the j-th indicator of the i-th object. The larger the value of RSR_i_, the better the evaluation object.

(4) Calculate the probit

After ranking, an RSR frequency distribution table is generated. This table lists the frequency f of each group in detail. At the same time, the cumulative frequency cf and cumulative frequency of each group are calculated. Finally, these values are converted into probit values.

(5) Calculate the linear regression equation

Consider the probit value as the independent variable and RSR as the dependent variable to calculate the linear regression equation.

(6) Classification and ranking

Use the estimated value of RSR (WRSR) calculated by the regression equation to classify and rank the evaluation objects.

#### 3.3.2. Optimization of Process Parameters for Activated Rubber Powder-Modified Asphalt

In this paper, G*/sinδ, the failure temperature, viscosity, and the GTS were selected as evaluation indicators to optimize the process parameters of activated rubber asphalt. Among them, the rutting factor and failure temperature are positive indicators, while viscosity and the GTS are negative indicators. The statistical results of the test values of each evaluation indicator of activated rubber powder-modified asphalt are shown in Table 5. The data of each evaluation indicator in Table 5 are processed by normalizing the same-direction trend, and the results are shown in Table 7.

The rank-sum ratio of each type of index data is calculated and ranked according to Formulas (5)–(7), as shown in Table 8.

The RSR values are arranged in an ascending order, and the downward cumulative frequency is calculated. Then, based on the cumulative frequency, the corresponding probit value is searched for in the “Percentage–Probit Conversion Table”. This value represents the distribution of RSR under a specific cumulative frequency. The calculation results are shown in Table 9.

The linear regression equation of RSR is derived by using the probit value as the independent variable to calculate relevant variables. According to the analysis of the F-test results, the *p*-value is 0.00 ***, indicating a high significance level. We reject the null hypothesis that the regression coefficient is 0. At the same time, the goodness-of-fit *R*^2^ of the model is 0.923, suggesting that the model performs well. Regarding variable collinearity, since all VIF values are less than 10, the model has no serious multicollinearity problem, and the model is well constructed. The model formula is as follows:

*RSR* = −0.005 + 0.102 × *Probit* (8)

The probit values of each sample are substituted into the regression model to calculate the fitted RSR values. Then, these fitted values are ranked from the largest to smallest to obtain the final ranking of the samples. The ranking of each sample is shown in Table 10.

As can be seen from Table 10, based on the comprehensive performance analysis, the top six samples are 7#-4, 7#-3, 3#-2, 6#-4, 6#-3, and 6#-1, i.e., the comprehensive performance of activated rubber asphalt is the best when the temperature is 220 °C, the reaction rate is 3000 rpm, and the mixing time is 240 min. The results show that the most dominant factor that affects the performance of activated rubber asphalt is temperature. When the reaction temperature of activated rubber asphalt is 220 °C, the comprehensive performance of activated rubber asphalt is obviously better than that at 190 °C and 160 °C. For this reason, the preparation temperature of activated rubber asphalt was finally selected as 220 °C. It is evident from Section 3.3 that stirring time and stirring rate have a lesser effect on the performance of rubber asphalt, and the rankings of 7#-4, 7#-3, 6#-4, 6#-3, and 6#-1 are all high. Therefore, the reaction time of activated rubber asphalt should be ≥120 min, and the reaction rate should be between 1000 and 3000 rpm.

In summary, taking into account economics, energy consumption, and other factors, the production process parameters of activated rubber asphalt that were finally selected in this study are as follows: a preparation temperature of 220 °C, a reaction time of 120 min and a stirring rate of 1000 rpm.

## 4. Conclusions

(1) Reaction temperature, stirring rate, and reaction time significantly affect the rheological properties of activated rubber asphalt. Among these parameters, reaction temperature exerts the most substantial influence, showing the highest gray correlation degree (>0.85) with asphalt performance indicators, followed by stirring rate and reaction time.
(2) Optimal parameters identified in this study are as follows: a reaction temperature of 220 °C, a stirring rate of 1000 rpm, and a reaction time of 120 min. Under these conditions, activated rubber asphalt exhibited superior high-temperature performance, reduced temperature sensitivity, and improved rheological stability.
(3) The rank-sum ratio (RSR) model combined with gray correlation analysis effectively integrates multiple rheological indexes, providing a comprehensive and objective evaluation of asphalt performance. This integrated evaluation approach successfully overcomes the limitations of single-indicator assessments, enhancing reliability and applicability in practical engineering.
(4) Future research is recommended to verify the long-term durability and fatigue resistance of the activated rubber asphalt produced under optimal parameters, promoting its broader applications in sustainable road construction.

## Figures and Tables

**Table 1:** Main technical indexes of virgin asphalt.

Test Item		SK90#	Specification Limits	Test Method
Penetration (25 °C, 100 g, 5 s)		92.2	80~100	T 0604
Softening point/°C		46.2	≥45	T 0605
Ductility (15 °C, 5 cm/min)		>100	≥100	T 0606
After RTFOT	Quality change (%)	0.07	≤±0.8	0.04
	Residual penetration ratio (25 °C) (%)	70	≥57	67.2
	Residual ductility (5 °C) (cm)	59.0	≥20	11.4

**Table 2:** Test results of physical indexes of waste tire rubber powder.

Test Item	Bulk Density (kg/m^3^)	Moisture/%	Metal Content/%	Fiber Content/%
Technical index	260–460	<1	<0.03	<1
Test result	305.6	0	0.011	0.076

**Table 3:** Test results of chemical indexes of waste tire rubber powder.

Test Item	Ash Content/%	Heating Loss/%	Acetone Extract/%	Carbon Black Content/%	Rubber Hydrocarbon Content/%
Technical index	≤8	≤1.0	≤22	≥28	≥42
Test result	7.5	0.76	7.5	30	49

**Table 4:** Design of production process parameters of activated rubber asphalt.

No.	CRM (%)	Stirring Rate (r/min)	Temperature (°C)	Time (min)
1#	10	1000	160	15
	10	1000	160	60
	10	1000	160	120
	10	1000	160	240
2#	10	2000	160	15
	10	2000	160	60
	10	2000	160	120
	10	2000	160	240
3#	10	1000	190	15
	10	1000	190	60
	10	1000	190	120
	10	1000	190	240
4#	10	2000	190	15
	10	2000	190	60
	10	2000	190	120
	10	2000	190	240
5#	10	2000	190	15
	10	2000	190	60
	10	3000	190	120
	10	3000	190	240
6#	10	3000	220	15
	10	3000	220	60
	10	1000	220	120
	10	1000	220	240
7#	10	1000	220	15
	10	1000	220	60
	10	3000	220	120
	10	3000	220	240

**Table 5:** Test values of different evaluation indexes of activated rubber asphalt.

No.	Time (min)	Stirring Rate (r/min)	Temperature (°C)	Viscosity/kPa·s	G*/sinδ/kPa	GTS	Failure Temperature/°C
1#-1	15	1000	160	0.224	1.4	5.2835	73.51
1#-2	60	1000	160	0.225	1.663	5.0891	75.06
1#-3	120	1000	160	0.251	1.792	4.9462	75.85
1#-4	240	1000	160	0.271	1.95	4.8456	76.7
2#-1	15	2000	160	0.251	1.694	5.0486	76.34
2#-2	60	2000	160	0.237	2.001	4.7611	75.22
2#-3	120	2000	160	0.26	1.914	4.9698	76.76
2#-4	240	2000	160	0.279	2.014	4.9353	76.88
3#-1	15	1000	190	0.292	1.778	4.8882	75.81
3#-2	60	1000	190	0.31	1.987	4.5079	76.59
3#-3	120	1000	190	0.327	1.927	4.602	76.7
3#-4	240	1000	190	0.342	1.809	4.4642	76.41
4#-1	15	2000	190	0.295	1.798	4.8335	75.68
4#-2	60	2000	190	0.305	2.002	4.7711	76.9
4#-3	120	2000	190	0.311	1.932	4.6937	76.63
4#-4	240	2000	190	0.36	1.866	4.4284	76.63
5#-1	15	3000	190	0.295	1.817	4.8514	76.02
5#-2	60	3000	190	0.351	2.044	4.4594	77.49
5#-3	120	3000	190	0.349	1.959	4.5876	76.77
5#-4	240	3000	190	0.365	1.652	4.4909	75.62
6#-1	15	1000	220	0.335	1.968	4.611	76.82
6#-2	60	1000	220	0.394	1.936	4.4347	77.1
6#-3	120	1000	220	0.281	2.073	4.7318	77.29
6#-4	240	1000	220	0.242	2.497	5.0272	78.1
7#-1	15	3000	220	0.374	1.694	4.556	75.68
7#-2	60	3000	220	0.256	1.535	4.9476	74.42
7#-3	120	3000	220	0.229	2.497	5.0272	78.19
7#-4	240	3000	220	0.23	3.023	4.9411	79.85

**Table 6:** Gray correlation degree value of each evaluation index and reaction parameter.

Parameters	Viscosity	G*/sinδ	GTS	Failure Temperature
Temperature	0.8638	0.8686	0.8539	0.8851
Stirring rate	0.6738	0.6804	0.6563	0.6409
Time	0.5754	0.5863	0.5585	0.5492

**Table 7:** Data matrix transformation results.

No.	X1: G*/sinδ/kPa	X2: Failure Temperature/°C	X3: Viscosity/kPa·s	X4: GTS
1#-1	0.00006	0.00002	0.99941	0.00012
1#-2	0.16209	0.24449	0.99354	0.22741
1#-3	0.24156	0.36909	0.84078	0.39448
1#-4	0.33890	0.50315	0.72327	0.51210
2#-1	0.18119	0.44637	0.84078	0.27476
2#-2	0.37032	0.26972	0.92303	0.61090
2#-3	0.31672	0.51262	0.78790	0.36689
2#-4	0.37833	0.53154	0.67626	0.40723
3#-1	0.23293	0.36278	0.59988	0.46229
3#-2	0.36169	0.48580	0.49412	0.90693
3#-3	0.32473	0.50315	0.39424	0.79691
3#-4	0.25203	0.45741	0.30611	0.95803
4#-1	0.24526	0.34228	0.58226	0.52625
4#-2	0.37093	0.53470	0.52350	0.59920
4#-3	0.32781	0.49211	0.48825	0.68970
4#-4	0.28715	0.49211	0.20035	0.99988
5#-1	0.25696	0.39590	0.58226	0.50532
5#-2	0.39681	0.62776	0.25323	0.96364
5#-3	0.34444	0.51420	0.26498	0.81375
5#-4	0.15531	0.33281	0.17098	0.92681
6#-1	0.34999	0.52208	0.34724	0.78639
6#-2	0.33027	0.56624	0.00059	0.99252
6#-3	0.41467	0.59621	0.66451	0.64515
6#-4	0.67589	0.72397	0.89365	0.29978
7#-1	0.18119	0.34228	0.11810	0.85070
7#-2	0.08323	0.14354	0.81140	0.39284
7#-3	0.67589	0.73816	0.97004	0.29978
7#-4	0.99994	0.99998	0.96416	0.40044

**Table 8:** The rank value of the data matrix calculates the ranking result.

No.	R1	R2	R3	R4	RSR	Rank
1#-1	1.00166	1.00043	27.98414	1.00316	0.27669	28
1#-2	5.37636	7.60116	27.82550	7.13995	0.42806	24
1#-3	7.52212	10.96541	23.70094	11.65100	0.48071	19
1#-4	10.15026	14.58517	20.52820	14.82673	0.53652	8
2#-1	5.89200	13.05210	23.70094	8.41845	0.45592	20
2#-2	10.99858	8.28253	25.92186	17.49421	0.55980	7
2#-3	9.55144	14.84068	22.27321	10.90600	0.51403	14
2#-4	11.21482	15.35171	19.25911	11.99509	0.51626	13
3#-1	7.28924	10.79507	17.19683	13.48194	0.43538	23
3#-2	10.76571	14.11673	14.34136	25.48720	0.57778	5
3#-3	9.76768	14.58517	11.64454	22.51666	0.52245	10
3#-4	7.80489	13.35019	9.26498	26.86671	0.51149	16
4#-1	7.62192	10.24146	16.72092	15.20870	0.44458	22
4#-2	11.01522	15.43688	15.13455	17.17853	0.52469	9
4#-3	9.85085	14.28707	14.18273	19.62189	0.51734	12
4#-4	8.75302	14.28707	6.40952	27.99684	0.51291	15
5#-1	7.93796	11.68936	16.72092	14.64363	0.45528	21
5#-2	11.71384	17.94942	7.83725	27.01824	0.57606	6
5#-3	10.29996	14.88327	8.15452	22.97124	0.50276	17
5#-4	5.19338	9.98595	5.61633	26.02385	0.41803	25
6#-1	10.44967	15.09620	10.37544	22.23255	0.51923	11
6#-2	9.91739	16.28859	1.01586	27.79797	0.49125	18
6#-3	12.19622	17.09771	18.94183	18.41915	0.59513	4
6#-4	19.24895	20.54713	25.12867	9.09400	0.66088	3
7#-1	5.89200	10.24146	4.18860	23.96878	0.39545	26
7#-2	3.24723	4.87570	22.90776	11.60680	0.38069	27
7#-3	19.24895	20.93040	27.19095	9.09400	0.68272	2
7#-4	27.99834	27.99957	27.03231	11.81200	0.84681	1

**Table 9:** Distribution table of RSR.

No.	RSR	Frequence	Cumulative Frequency Σf	Evaluation Rank Number	Evaluation Rank Number/*n*00%	Probit
1#-1	0.27669	1	1	1	3.57143	3.19726
7#-2	0.38069	1	2	2	7.14286	3.53477
7#-1	0.39545	1	3	3	10.71429	3.75813
5#-4	0.41803	1	4	4	14.28571	3.93243
1#-2	0.42806	1	5	5	17.85714	4.07918
3#-1	0.43538	1	6	6	21.42857	4.20836
4#-1	0.44458	1	7	7	25.00000	4.32551
5#-1	0.45528	1	8	8	28.57143	4.43405
2#-1	0.45592	1	9	9	32.14286	4.53629
1#-3	0.48071	1	10	10	35.71429	4.63389
6#-2	0.49125	1	11	11	39.28571	4.72812
5#-3	0.50276	1	12	12	42.85714	4.81999
3#-4	0.51149	1	13	13	46.42857	4.91036
4#-4	0.51291	1	14	14	50.00000	5.00000
2#-3	0.51403	1	15	15	53.57143	5.08964
2#-4	0.51626	1	16	16	57.14286	5.18001
4#-3	0.51734	1	17	17	60.71429	5.27188
6#-1	0.51923	1	18	18	64.28571	5.36611
3#-3	0.52245	1	19	19	67.85714	5.46371
4#-2	0.52469	1	20	20	71.42857	5.56595
1#-4	0.53652	1	21	21	75.00000	5.67449
2#-2	0.55980	1	22	22	78.57143	5.79164
5#-2	0.57606	1	23	23	82.14286	5.92082
3#-2	0.57778	1	24	24	85.71429	6.06757
6#-3	0.59513	1	25	25	89.28571	6.24187
6#-4	0.66088	1	26	26	92.85714	6.46523
7#-3	0.68272	1	27	27	96.42857	6.80274
7#-4	0.84681	1	28	28	99.10714	7.36857

**Table 10:** Statistical results of sorting of each sample.

No.	Stirring Rate (r/min)	Temperature (°C)	Time (min)	Rank	Probit	Fitted Value	Final Ranking
7#-4	3000	220	240	1	7.36857	0.74432	1
7#-3	3000	220	120	2	6.80274	0.68679	2
3#-2	1000	190	60	5	6.06757	0.61205	3
6#-4	1000	220	240	3	6.46523	0.65248	4
6#-3	1000	220	120	4	6.24187	0.62977	5
6#-1	1000	220	15	11	5.36611	0.54073	6
5#-2	3000	190	60	6	5.92082	0.59713	7
4#-2	2000	190	60	9	5.56595	0.56105	8
3#-3	1000	190	120	10	5.46371	0.55065	9
4#-3	2000	190	120	12	5.27188	0.53115	10
2#-2	2000	160	60	7	5.79164	0.58399	11
2#-4	2000	160	240	13	5.18001	0.52181	12
1#-4	1000	160	240	8	5.67449	0.57208	13
2#-3	2000	160	120	14	5.08964	0.51262	14
3#-1	1000	190	15	23	4.20836	0.42301	15
1#-3	1000	160	120	19	4.63389	0.46628	16
7#-2	3000	220	60	27	3.53477	0.35453	17
7#-1	3000	220	15	26	3.75813	0.37724	18
2#-1	2000	160	15	20	4.53629	0.45636	19
6#-2	1000	220	60	18	4.72812	0.47586	20
5#-4	3000	190	240	25	3.93243	0.39496	21
3#-4	1000	190	240	16	4.91036	0.49439	22
5#-3	3000	190	120	17	4.81999	0.48520	23
5#-1	3000	190	15	21	4.43405	0.44596	24
4#-4	2000	190	240	15	5.00000	0.50350	25
1#-2	1000	160	60	24	4.07918	0.40988	26
4#-1	2000	190	15	22	4.32551	0.43493	27
1#-1	1000	160	15	28	3.19726	0.32021	28

## Data Availability

The original contributions presented in this study are included in the article. Further inquiries can be directed to the corresponding authors.
